# Applications and Benefits of Dietary Supplements in Taekwondo: A Systematic Review

**DOI:** 10.3390/life15040559

**Published:** 2025-03-29

**Authors:** Meng-Yuan Shu, Jian Liang, Young-Jin Jo, Seon-Ho Eom, Chul-Hyun Kim

**Affiliations:** 1 Department of Sports Medicine, Soonchunhyang University, Asan 31538, Republic of Korea; 2 Department of Biology, Soonchunhyang University, Asan 31538, Republic of Korea

**Keywords:** taekwondo, dietary supplements, adverse reactions, performance

## Abstract

Dietary supplements are commonly employed to provide additional nutritional support for athletes. In taekwondo, there is a need for evidence-based analyses to evaluate the effects of dietary supplements on training outcomes, competitive performance, and injury recovery. Taekwondo primarily relies on oxidative metabolism, yet decisive lower-limb attacks depend on non-oxidative pathways. A comprehensive literature search was conducted in PubMed, Scopus, and Web of Science databases in November 2024, utilizing keywords including ‘Dietary Supplements’, ‘Supplements’, ‘Food Supplementations’, and ‘Taekwondo’. Of the 203 articles identified, 26 met the inclusion criteria, collectively assessing the impact of 14 different dietary supplements. Among these studies, eight provided strong evidence that acute ingestion of 3–5 mg/kg of caffeine significantly enhanced athletes’ physical performance and psychological well-being. However, the ergogenic effects of the compound Fufang Ejiaojiang, creatine, sodium bicarbonate, beetroot, vitamins, and long beans require further investigation to validate their efficacy. Additionally, dietary supplements, such as amino acids, turmeric powder, ginger, spirulina, octacosanol, nucleotides, and yogurt, can reduce fatigue, supporting injury recovery, and boosting immune function, although current evidence remains insufficient. Future research should pay closer attention to reporting adverse reactions linked to dietary supplements. Doing so would provide coaches and athletes with more reliable safety information, supporting safer choices and reducing potential health risks.

## 1. Introduction

Taekwondo, a sport that primarily uses the feet for combat or competition, originated during the Three Kingdoms period on the Korean Peninsula [1]. Since its inclusion as an official event in the 2000 Sydney Olympics, taekwondo has gained increasing attention worldwide. As a combat sport, taekwondo is characterized by short bursts of intense movements interspersed with more extended periods of low-intensity actions [2]. Oxidative metabolism is the primary energy pathway during taekwondo combat; however, lower-limb attacks driven by non-oxidative metabolism are important in determining match outcomes [2]. Additionally, the high-intensity nature of taekwondo frequently leads to muscle damage and immune suppression during competitions and regular training sessions [3]. Consequently, significant research has focused on using dietary supplements as part of taekwondo athletes’ nutritional strategies to enhance athletic performance and support recovery from sports-related injuries.

Dietary supplements are intended to complement regular diets and commonly include vitamins, minerals, natural herbs, creatine, and other substances with potential benefits for health and athletic performance. These supplements have gained significant popularity among athletes and are often integrated into their regular training and competition routines [4]. Although numerous review studies have examined the use of dietary supplements in combat sports [5,6], their specific application in taekwondo remains unexplored. For instance, although caffeine has been shown to enhance performance across combat sports [5], the strength requirements vary significantly, with boxing emphasizing upper-limb strength, karate involving both upper- and lower-limb strength and taekwondo emphasizing lower-limb strength [7,8,9]. Furthermore, different combat sports subject athletes to distinct types and degrees of physical injuries [10]. Additionally, existing reviews have predominantly focused on the performance-enhancing effects of dietary supplements [11,12], often overlooking critical aspects relevant to taekwondo athletes, such as alleviating muscle damage, reducing psychological fatigue, and facilitating immune recovery. Thus, a comprehensive review specifically addressing dietary supplements in taekwondo is necessary to provide targeted guidance and evidence-based recommendations for athletes and coaches.

Given these unique physiological demands and injury characteristics, it is important to clarify how dietary supplements specifically affect taekwondo athletes. Although supplements may offer potential benefits, their effectiveness could vary depending on an athlete’s skill level and sex [13], and they may also have potential side effects [14]. Therefore, this systematic review aims to evaluate current evidence regarding dietary supplement use in taekwondo, emphasizing its effects on athletic performance and recovery from sports injuries, to provide practical and evidence-based recommendations for athletes and coaches.

## 2. Materials and Methods

This systematic review adheres to the guidelines outlined in the Preferred Reporting Items for Systematic Reviews and Meta-Analyses (PRISMA) for its conduct and reporting [15] (Table S1). This study has been registered in the International Prospective Register of Systematic Reviews (PROSPERO) under the registration number CRD420251006028.

### 2.1. Literature Search and Selection of Studies

On 18 November 2024, two researchers (M.-Y.S. and J.L.) independently conducted English-language literature searches in the PubMed, Scopus, and Web of Science databases, with the results subsequently verified by a third researcher (C.-H.K.). The following keywords and Boolean phrases were used: Dietary Supplements OR Dietary Supplement OR Supplements, Dietary OR Dietary Supplementations OR Supplementations, Dietary OR Food Supplementations OR Food Supplements OR Food Supplement OR Supplement, Food OR Supplements, Food OR Nutraceuticals OR Nutraceutical OR Nutriceuticals OR Nutriceutical OR Neutraceuticals OR Neutraceutical OR Herbal Supplements OR Herbal Supplement OR Supplement, Herbal OR Supplements, Herbal OR Caffeine OR Creatine OR Beta-alanine OR Sodium Bicarbonate OR Nitrates OR Glycerol OR Vitamin OR Amino acid OR Beet OR Nitrate OR Protein OR Tea, AND Taekwondo.

The inclusion criteria were as follows: (1) studies involving taekwondo athletes aged over 15 years, regardless of sex and competitive level; (2) randomized controlled trials (RCTs) that compared a dietary supplement intervention group with a placebo or no-supplement control; (3) studies evaluating outcomes related to athletic performance, recovery from injury or muscle damage, fatigue, immune function, or psychological effects; (4) studies that did not involve substances prohibited by the World Anti-Doping Agency (WADA); and (5) studies published in English and not classified as gray literature.

### 2.2. Data Extraction

Two researchers (M.-Y.S. and J.L.) independently extracted the following information from each included study: (1) study authors and year; (2) type of dietary supplement; (3) dosage; (4) duration of intervention; (5) participants’ sex; (6) physical characteristics (e.g., age, height, weight); (7) competitive level; and (8) main outcomes related to athletic performance and recovery. Disagreements were resolved by consultation with a third researcher (C.-H.K.). These data variables were selected because supplement type and dosage are critical for understanding effectiveness and safety, intervention duration provides insight into supplement efficacy timelines, participant characteristics (e.g., sex, physical attributes, competitive level) can influence the response to supplementation, and outcome measures directly reflect supplement effectiveness regarding athletic performance and recovery.

### 2.3. Quality Assessment

The risk of bias in the included randomized controlled trials (RCTs) was assessed using the Cochrane collaboration’s tool for assessing the risk of bias in a randomized trial (RoB 1) [16]. The following criteria were analyzed: random sequence generation (selection bias), allocation concealment (selection bias), blinding of participants and personnel (performance bias), blinding of outcome assessment (detection bias), incomplete outcome data (attrition bias), selective reporting (reporting bias), and other biases.

Two researchers (M.-Y.S. and J.L.) independently found the risk of bias in the literature, with a third researcher (C.-H.K.) resolving any disagreements through consensus.

### 2.4. Data Analysis and Synthesis

The extracted data were descriptively analyzed and systematically summarized to identify key findings, clarify existing gaps in the literature, and offer practical guidance for taekwondo athletes, coaches, and trainers regarding the use of dietary supplements. This synthesis also establishes a foundation for future research aimed at safer and more effective dietary supplement practices in taekwondo.

## 3. Results

### 3.1. Included Studies and Study Characteristics

**Figure 1:**
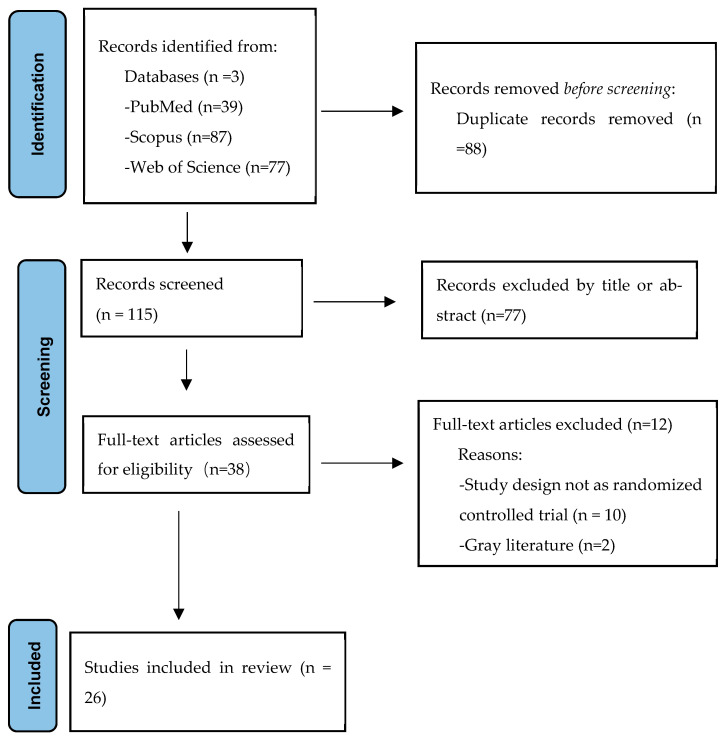
Flow chart for study inclusion and exclusion process based on PRISMA.

We retrieved 203 articles from three databases: PubMed (39), Scopus (87), and Web of Science (77). After eliminating 88 duplicate records, 77 articles were excluded based on their titles and abstracts. Following a thorough full-text review, 10 articles that did not meet the RCT criteria and two gray literature articles were excluded. Ultimately, this study included 26 articles (Figure 1).

Among the twenty-six studies, eight focused on evaluating the effectiveness of caffeine as a dietary supplement [13,17,18,19,20,21,22,23]. Additionally, three studies examined the role of vitamins [3,24,25], whereas another three investigated the effects of beetroot [26,27,28]. The remaining studies explored various supplements, including Branched-chain amino acids, carob, creatine, curcumin, Fufang Ejiaojiang, ginger, nucleotides, octacosanol, sodium bicarbonate, spirulina, and yogurt [29,30,31,32,33,34,35,36,37,38,39]. Notably, one study addressed the combined supplementation of creatine and sodium bicarbonate [40] (Table 1).

In total, 610 participants were involved in the 26 studies. Among them, the study by Wang et al. (2020) [33] included the highest sample size (90 participants), whereas that of Miraftabi et al. (2021) [28] had the smallest sample size (eight participants). The participants’ age, height, and weight ranges were 17–27 years, 152–187 cm, and 43–77.8 kg, respectively.

### 3.2. Risk of Bias

**Figure 2:**
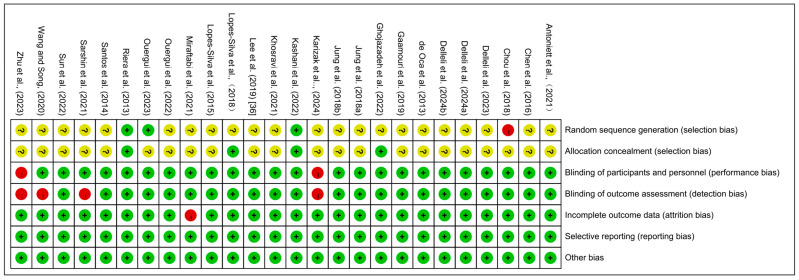
Risk of bias summary: review authors’ judgements about each risk of bias item for each included study [3,13,15,16,18,19,20,22,23,24,25,26,27,29,30,31,32,33,34,35,36,37,38,39,40].

**Figure 3:**
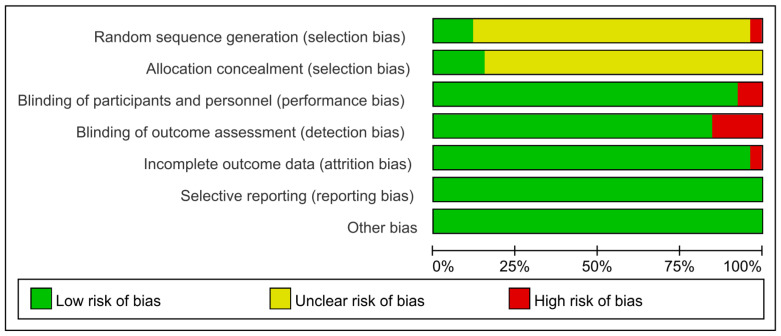
Risk of bias graph: review of authors’ judgements about each risk of bias item presented as percentages across all included studies.

The risk of bias in the included studies is illustrated in Figure 2 and Figure 3. Most of the trials assessed exhibited an unclear risk of bias in random sequence generation and allocation concealment. However, only the studies by Kashani et al. (2022) [38] and Riera et al. (2013) [35] explicitly detailed the criteria and procedures for these aspects. Notably, Chou et al. (2018) [3] employed a randomization process based on the athletes’ body weight, introducing a higher risk of bias in random sequence generation. Additionally, in the study by Miraftabi et al. (2021) [28], four out of twelve athletes could not complete the experiment due to injuries, resulting in a high risk of bias in incomplete outcome data. Furthermore, four studies demonstrated a high risk of bias in blinding participants and personnel and blinding of outcome assessment, primarily due to incomplete or absent blinding procedures [34,39,40].

### 3.3. Caffeine

Among the eight studies on caffeine, two administered a dosage of 5 mg/kg [17,18], one employed a single dose of 200 mg [14], and the remaining studies administered a dosage of 3 mg/kg [13,19,21,22,23].

In Santos et al. (2014) [17], 10 athletes with seven years of Taekwondo training experience consumed 5 mg/kg of caffeine, significantly reducing reaction time and improving performance in simulated Taekwondo matches. Conversely, Lopes-Silva et al. (2015) [18] investigated 10 athletes with nine years of training experience who consumed the same dosage of caffeine. Although caffeine increased glycolytic metabolism during simulated sparring, it did not enhance performance or parasympathetic activation. Similarly, Sun et al. (2022) [20] studied 10 athletes with five years of training experience who consumed a single dose of 200 mg of caffeine. The results showed improved anaerobic capacity but no significant improvements in performance during simulated matches.

Moreover, Ouergui et al. (2023) [13] conducted a study with 52 elite and sub-elite athletes consuming 3 mg/kg of caffeine, demonstrating that caffeine’s ergogenic effects were more pronounced in men than women and elite athletes than sub-elite athletes. Similarly, in Ouergui et al. (2022) [19], 20 athletes with six years of training experience consumed 3 mg/kg of caffeine combined with training activities. The results revealed that this combination was the most effective in enhancing athletic performance, with men outperforming women in physical and psychological performance.

Delleli et al. (2024a) [22] and Delleli et al. (2024b) [23] focused on the combined effects of 3 mg/kg of caffeine and listening to music on Taekwondo athletes. Male athletes who consumed caffeine while listening to music exhibited the best athletic performance and reported higher physical enjoyment than other groups [21]. Contrastingly, female athletes consuming caffeine and listening to music demonstrated the best athletic performance, the lowest average and peak heart rates, and optimal psychological state [22]. This combination was shown to be the most effective strategy for enhancing athletic performance and promoting positive emotions in female athletes [23].

### 3.4. Vitamin

Among the studies included in this systematic review, three specifically investigated the effects of vitamins. Chou et al. (2018) [3] examined the impact of consuming 2000 mg/day of vitamin C and 1400 IU/day of vitamin E on reducing muscle damage and inflammatory responses in 18 male athletes. The results demonstrated that the vitamin supplementation group had significantly lower levels of myoglobin (*p* = 0.021), plasma creatine kinase (*p* = 0.017), and hemolysis (*p* = 0.034) compared to the placebo group.

In Jung et al. (2018a) [24], 44 vitamin-D-deficient collegiate Taekwondo athletes were supplemented with 5000 IU/day of vitamin D during the winter. The findings revealed improvements in anaerobic peak power and isokinetic muscle strength in the vitamin D supplementation group. Similarly, Jung et al. (2018b) [25] investigated 26 vitamin-D-deficient male collegiate Taekwondo athletes during the winter and found that supplementation with 5000 IU/day of vitamin D significantly reduced salivary lactoferrin levels (*p* = 0.011) in the supplementation group.

### 3.5. Creatine and Sodium Bicarbonate

Manjarrez-Montes de Oca et al. (2013) [31] investigated the effects of 50 mg/kg of creatine on 10 male Taekwondo practitioners, revealing that supplementation increased fat mass (*p* < 0.028) and serum triglyceride concentrations (*p* < 0.037) but had no effect on anaerobic capacity. Lopes-Silva et al. (2018) [37] evaluated the impact of 300 mg/kg of sodium bicarbonate on the performance of nine elite athletes. The results showed that sodium bicarbonate supplementation led to higher lactate concentrations (*p* = 0.04), greater glycolytic energy contributions (*p* = 0.01), and a significant increase in total attack time during matches (*p* = 0.05). Expanding on the interplay between these supplements, Sarshin et al. (2021) [40] examined the combined effects of creatine and sodium bicarbonate supplementation on anaerobic performance in 40 male elite Taekwondo athletes. Their findings indicated that both creatine and sodium bicarbonate, when taken individually, improved performance in the Taekwondo Anaerobic Intermittent Kick Test, and their combined use further enhanced mean power.

### 3.6. Herbal Supplements

Three studies focused on the effects of beetroot supplementation. In the study of Antoniett et al. (2021) [26], 12 male athletes consuming 1 g of beetroot extract demonstrated significantly higher VO_2_ peak (*p* = 0.048) and anaerobic threshold (*p* = 0.044) compared to the placebo group. The other two studies examined the effects of beetroot juice. In Khosravi et al. (2021) [27], 12 male athletes consuming 120 mL of beetroot juice exhibited greater knee extensor peak torque at 180°/s and 360°/s angular velocities for the dominant leg and higher peak torque at 360°/s for the non-dominant leg. Miraftabi et al. (2021) [28] evaluated the effects of 60 mL and 120 mL of beetroot juice on 12 male athletes. The results showed that the 60 mL group had significantly better cognitive function (*p* < 0.05) than the placebo and 120 mL groups.

Carob contains polyphenols that can positively influence athletes’ energy metabolism and antioxidant capacity. Gaamouri et al. (2019) [30] examined the effects of consuming 40 g of carob daily over six weeks in 23 athletes. Compared to the placebo group, the supplementation group showed a significant reduction in body weight (*p* < 0.001) and BMI (*p* < 0.001). Additionally, the supplementation group demonstrated notable improvements in total exercise distance (*p* < 0.001), maximum aerobic speed (*p* < 0.001), and perceived exertion (*p* < 0.000).

Curcumin, a natural herbal compound extracted from turmeric, is known for its anti-inflammatory and antioxidant properties [41]. Ghojazadeh et al. (2022) [32] evaluated the physiological indicators of 18 male athletes who consumed 4 g/day of turmeric powder for six days. Compared to the placebo group, the supplementation group exhibited significantly lower levels of creatine kinase (*p* < 0.05), lactate dehydrogenase (*p* < 0.05), and malondialdehyde (*p* < 0.05).

The primary component of Fufang Ejiaojiang is donkey-hide gelatin (Ejiao), a traditional tonic in Chinese medicine [42]. A study evaluated the long-term effects of Fufang Ejiaojiang supplementation on 90 athletes. Compared to the placebo group, the supplementation group demonstrated significantly higher levels of superoxide dismutase, catalase, glutathione peroxidase, hydroxyl radical, malondialdehyde, and total antioxidant capacity (*p* < 0.05). Additionally, the levels of leukocytes, hemoglobin, and hematocrit were significantly increased. Regarding performance, the relative maximum oxygen uptake, peak anaerobic power, and mean anaerobic power were significantly improved (*p* < 0.05), along with enhanced back strength, grip strength, squat strength, and bench press strength (*p* < 0.05). Furthermore, the levels of creatine kinase and blood urea nitrogen were significantly reduced (*p* < 0.05) [33].

The medicinal value of ginger has been widely validated. For instance, consuming fresh ginger has been shown to alleviate post-exercise pain [43]. A study evaluated the effects of consuming 2 g of ginger daily on reducing post-exercise inflammation and muscle soreness in 24 semi-professional female athletes. Compared to athletes who did not consume ginger, the supplementation group exhibited significantly lower levels of serum prostaglandin E2 (*p* < 0.001), cyclooxygenase-2 (*p* = 0.003), and interleukin-6 (*p* = 0.006) [34].

Spirulina, known for its high protein content, exhibits antioxidant and anti-inflammatory properties, making it a widely used daily supplement among athletes [44]. Kashani et al. (2022) [38] investigated the biochemical changes in 18 male athletes who consumed 8 g of spirulina daily for three weeks. Compared to the placebo group, the spirulina supplementation group showed significantly lower plasma lactate dehydrogenase levels, creatine kinase, and interleukin-6 (*p* < 0.05). Conversely, plasma total antioxidant capacity levels, superoxide dismutase, and glutathione peroxidase significantly increased (*p* < 0.05).

### 3.7. Amino Acids, Nucleotide, Octacosanol, and Yogurt

Exercise-induced central fatigue can be alleviated by consuming branched-chain amino acids [45]. A study evaluated the effects of branched-chain amino acids (BCAAs) on alleviating central fatigue in 12 male Taekwondo athletes. Compared to the placebo group, the BCAA supplementation group maintained their reaction time after three simulated matches and exhibited significantly higher NOx concentrations (*p* = 0.049) [29].

Nucleotides are crucial in most biochemical processes. Research has indicated that dietary nucleotides positively impact the immune system [46]. Riera et al. (2013) [35] evaluated the effects of 480 mg of Inmunactive supplementation daily for 30 days on immune function markers in 20 elite male Taekwondo athletes following intense exercise under cold conditions. The results showed that the supplementation group recovered more quickly from the lymphocyte reduction response than the placebo group (*p* = 0.0028) and exhibited a significantly higher lymphocyte proliferation response (*p* = 0.0045).

Octacosanol is a naturally occurring long-chain fatty alcohol widely found in nature. Research has shown that it offers various metabolic and cardiovascular health benefits [47]. A study evaluated the physiological effects of 40 mg/day of octacosanol supplementation over six days in 26 male Taekwondo athletes undergoing a 5% weight reduction program combined with Taekwondo training. Compared to the placebo group, the supplementation group showed a significant decrease in low-density lipoprotein by 18 ± 5 mg/dL and triglycerides (TG) by 80 ± 7 mg/dL (*p* < 0.05), whereas high-density lipoprotein significantly increased by 10 ± 7 mg/dL, (*p* < 0.05). Additionally, superoxide dismutase levels significantly increased at 226 ± 121 U/gHb (*p* < 0.05) [36].

Yogurt typically contains a high concentration of probiotics, which can alter gut microbiota composition [48]. Some studies have suggested that gut microbiota may impact cognitive function and emotional well-being [49,50]. In a study by Zhu et al. (2023) [39], the effects of 8 weeks of yogurt consumption on gut microbiota and exercise-related psychological fatigue were evaluated in 51 female Taekwondo athletes. The results showed that the gut microbiota composition of athletes consuming yogurt differed significantly from that of the control group. Additionally, the athletes in the yogurt group scored significantly higher on the Athlete Burnout Questionnaire than the control group (*p* < 0.05).

## 4. Discussion

### 4.1. Impact of Dietary Supplements on Taekwondo Performance

Caffeine, widely recognized as a supplement that enhances aerobic and anaerobic performance [51], has been extensively adopted in various sports activities since its removal from the list of prohibited stimulants in 2004 [52]. Caffeine modulates central nervous system function by suppressing parasympathetic nervous system activity, increasing alertness and reducing fatigue perception [53]. It also elevates blood norepinephrine levels, stimulating glycolysis, enhancing muscle energy availability and sustaining performance during exercise [54]. Among the 26 selected studies, caffeine emerged as the most extensively researched supplement, with most studies focusing on intake levels of 5 mg/kg, reporting significant psychological and physiological benefits. Three studies reported that pre-competition caffeine consumption enhances athletes’ anaerobic capacity, resulting in improvements in reaction time, peak power, agility, and kicking speed [13,17,20]. However, one study found that, despite the observed improvements in anaerobic capacity, caffeine intake did not lead to better overall athletic performance [18]. Furthermore, differences in athletic level and sex were found to influence the effects of caffeine on athletic performance under the same intake conditions [13]. Studies suggest that caffeine metabolism is influenced by genetic factors, particularly the CYP1A2 and ADORA2A enzymes, which regulate its absorption and breakdown [55]. Female taekwondo athletes, in particular, tend to metabolize caffeine more slowly than men, a difference that becomes more pronounced during the menstrual cycle due to hormonal fluctuations affecting caffeine clearance and physiological response [13]. Although there are pharmacokinetic differences between men and women, research indicates that moderate acute doses of caffeine (3–6 mg/kg) lead to similar improvements in strength and endurance performance in both sexes. There is little evidence to support sex-specific dosing strategies [56]. Studies involving women have shown that a dose of around 6 mg/kg can enhance both strength and aerobic capacity [56]. Due to a generally smaller body size and slower metabolism, women may reach optimal arousal at slightly lower absolute doses. Nonetheless, the same relative dose range is effective for both men and women. When planning caffeine use, women should also consider the timing of their menstrual cycle and the potential for increased sensitivity. All studies confirmed the positive effects of caffeine consumption compared to placebo or non-caffeine groups. Notably, combining caffeine intake with listening to music or engaging in conditioning activities further enhanced athletes’ psychological and physiological performance [19,21,22,23]. Despite these findings, research on the combined effects of caffeine with other supplements in Taekwondo athletes remains limited. Future studies could explore the potential benefits of combining caffeine with sodium bicarbonate or caffeine with carbohydrates. Additionally, further research is needed to investigate the physiological and psychological effects of high-dose caffeine consumption on Taekwondo athletes to optimize supplementation strategies.

Beetroot is rich in inorganic nitrate, and numerous studies have demonstrated that its consumption can enhance endurance performance, improve cognitive function, and reduce reaction time in athletes [57,58,59]. While the short-term ergogenic effects of beetroot supplementation have been widely validated, its acute effects remain inconsistent [28]. For instance, Miraftabi et al. (2021) [28] found that pre-competition consumption of 120 or 60 mL of beetroot juice did not enhance performance in male Taekwondo athletes. In contrast, Antoniett et al. (2021) [26] reported that acute pre-competition supplementation with 1 g of beetroot extract significantly improved aerobic capacity in the same athletic population. This discrepancy may stem from individual differences in nitrate metabolism. After ingestion, inorganic nitrate (NO_3_^−^) is first reduced to nitrite (NO_2_^−^) by anaerobic bacteria in the oral cavity, then further converted into nitric oxide (NO) in the stomach’s acidic environment [60]. This process triggers various physiological mechanisms that contribute to performance enhancement. However, variability in the composition of the oral microbiome, genetic polymorphisms related to nitrate metabolism, and differences in athletic conditioning or training level can lead to inconsistent research findings [61]. Also, higher inorganic nitrate concentrations may elicit more pronounced ergogenic effects [62].

Traditional Chinese medicine (TCM), despite its thousands of years of history, remains challenging to explain mechanistically due to its complex composition [63]. Nevertheless, some studies suggest that TCM can alleviate exercise-induced fatigue [64]. Fufang Ejiaojiang (FEJ), a traditional TCM preparation, contains donkey-hide collagen as its main component. A study evaluating Taekwondo athletes who consumed 60 mL of FEJ daily for two months reported significant improvements in antioxidant capacity, blood oxygen transport, aerobic capacity, and overall exercise performance, along with a reduction in exercise-induced fatigue [33]. These findings align with extensive research showing that oral collagen supplementation can positively influence athletes’ joint function, muscle recovery, and musculoskeletal system [65]. However, FEJ contains other herbal ingredients, such as codonopsis pilosula and rehmannia root, which may contribute to its overall effects. Therefore, future research is needed to isolate and independently evaluate the ergogenic effects of donkey-hide collagen better to understand its specific role in athletic performance and recovery.

Creatine and sodium bicarbonate work together by helping to buffer H^+^ ions inside and outside the cells, which helps maintain muscle pH balance [66]. Creatine supports adenosine triphosphate production and boosts power output, while sodium bicarbonate reduces acid buildup and extends high-intensity exercise performance [67]. This combination improves energy use, delays fatigue, and aids recovery, improving overall athletic performance [66,67]. Currently, only one study specifically examined the effects of this combination on anaerobic capacity in Taekwondo athletes [40]. Athletes consumed 0.5 g/kg of sodium bicarbonate and 20 g/day of creatine for five days. The athletes achieved a significantly higher power output and lower blood lactate levels after specific tests than the control group. Moreover, combined supplementation showed a clear advantage in mean power output compared to creatine or sodium bicarbonate alone. Similarly, in another experiment, Taekwondo athletes who consumed 0.3 g/kg of sodium bicarbonate 90 min before competition exhibited higher blood lactate concentrations, indicating an increased contribution from glycolytic energy production and a significant increase in total attack time compared to the placebo group [37]. A separate study in which Taekwondo practitioners supplemented with 50 mg/kg of creatine for six weeks found no improvement in anaerobic capacity [33]. These findings indicate that acute sodium bicarbonate supplementation can enhance athletic performance, whereas the athlete’s competitive level may influence the ergogenic effects of creatine supplementation.

Vitamin D deficiency is common among professional athletes, and insufficient vitamin D levels may lead to reduced muscle strength and fatty degeneration of type II muscle fibers, ultimately impacting athletic performance [68]. Taekwondo athletes typically train indoors, which increases the likelihood of vitamin D deficiency compared to athletes in other sports [69]. In a study involving Taekwondo athletes with vitamin D deficiency, a four-week vitamin D supplementation resulted in modest improvements in specific physical performance measures in the supplementation group compared to the placebo group. Notably, the supplementation group exhibited a lower injury rate than the placebo group [25]. Given the indoor training environment and physical demands of Taekwondo, regular monitoring of serum 25(OH)D concentrations is recommended to ensure optimal performance and reduce the risk of injury in taekwondo athletes.

Carob is abundant in polyphenols, which have been shown to positively influence energy metabolism and possess antioxidant properties [70]. After six weeks of daily supplementation with 40 g of carob (200 mg polyphenols), Taekwondo athletes in the supplementation group showed a reduction in body weight and improved aerobic performance [30]. Notably, the ergogenic effects of polyphenols can vary depending on the population and dosage [71].

Overall, the ergogenic effects of caffeine, beetroot, carob, and sodium bicarbonate in Taekwondo athletes vary depending on their performance level. This variation may be attributed to differences in energy metabolism and substrate utilization, neuromuscular adaptations, and body composition between athletes of different training statuses. Furthermore, considering physiological differences between males and females, future research should focus on exploring sex-based differences in the ergogenic effects of various dietary supplements among Taekwondo athletes. This includes exploring how factors like enzyme activity, muscle fiber composition, and hormone-related metabolism may influence individual responses, helping to guide more targeted and effective supplementation strategies.

### 4.2. Role of Dietary Supplements in Recovery for Taekwondo Sports

Vitamins are essential for maintaining human health [72]. Vitamin D deficiency has been linked to an increased risk of upper respiratory tract infections (URTI) in professional athletes [73]. Research shows that taekwondo athletes with vitamin D deficiency experienced a significant reduction in URTI symptoms after daily supplementation with 5000 IU of vitamin D [25]. As a combat sport, taekwondo frequently results in muscle injuries due to its high-intensity nature. Natural antioxidants like vitamins C and E have been shown to mitigate exercise-induced muscle tissue damage [74]. Furthermore, studies indicate that elite taekwondo athletes who consumed 2000 mg of vitamin C and 1400 IU of vitamin E daily for four consecutive days experienced a marked decrease in exercise-induced tissue damage and inflammatory responses [3]. As vitamins are readily obtained from a balanced diet, we recommend offering taekwondo athletes vitamin-rich foods to ensure they meet their nutritional requirements.

Amino acids and nucleotides are essential biomolecules critical in various biochemical reactions [75]. The combined supplementation of BCAA, arginine, and citrulline has effectively reduced exercise-induced central fatigue in elite taekwondo athletes without leading to additional NH_3_ accumulation [29]. Although BCAA supplementation can reduce fatigue in athletes, it is associated with increased NH_3_ accumulation in vitro [76]. However, combined amino acid supplementation appears to mitigate this effect. Further research is needed to explore the potential roles of arginine and citrulline in reducing BCAA-induced NH_3_ accumulation. Besides amino acids, exogenous nucleotides have been found to stimulate the maturation of immune cells [77]. For taekwondo athletes undergoing high-intensity training, continuous supplementation with 480 mg of nucleotides over 30 days has proven effective in reducing training-induced immune damage [35].

Ginger and turmeric, though from the same family (Zingiberaceae), work differently as regards post-exercise recovery. Female taekwondo athletes taking 2 g of ginger daily saw a drop in IL-6 levels, likely because ginger directly inhibits COX enzymes, reducing inflammation, and estrogen has anti-inflammatory effects [34]. Meanwhile, male athletes who took 4 g of turmeric had higher IL-6 levels after exercise, possibly because curcumin temporarily boosts inflammation to help muscle adaptation [32]. On the other hand, those who were supplemented with 8 g of spirulina for 21 days had lower CK, LDH, and IL-6 levels, suggesting spirulina helps reduce exercise-induced muscle damage and inflammation, likely through its antioxidant properties [38].

Acute weight reduction before competitions is a widespread practice among taekwondo athletes, but it often poses significant risks to physical health [37]. Notably, during periods of rapid weight loss involving caloric restriction and high-intensity exercise, a six-day supplementation with 40 mg of octacosanol has been found to enhance lipid profiles and promote a healthier balance between antioxidant enzymes and oxidative stress markers in taekwondo athletes [36].

Taekwondo athletes often experience significant psychological stress during competitions. Research has shown that daily supplementation with up to 250 mL of yogurt over eight weeks can reduce exercise-induced psychological fatigue in female taekwondo athletes [39]. However, the effectiveness of probiotic supplementation is influenced by several external factors, including the dosage and duration, the specific probiotic formulation, and the efficacy of the strains used [78]. Future studies should prioritize high-quality experimental designs, such as double-blind trials with standardized dosages and controlled variables, to ensure more reliable and consistent outcomes.

### 4.3. Adverse Reactions of Dietary Supplements

Dietary supplements can be marketed in Europe and the United States without safety certification [79]. However, this does not mean they are inherently safe. Adverse reactions related to dietary supplement use are relatively common [79]. Interestingly, among the 26 studies included in our analysis, only five reported adverse reactions, primarily involving gastrointestinal discomfort and headaches [21,23,28,37,38]. Notably, 3 mg/kg caffeine supplementation was associated with more adverse effects in taekwondo athletes than other dietary supplements, including gastrointestinal discomfort and headaches. However, no adverse reactions were reported in other studies investigating caffeine supplementation at 5 mg/kg [17,18]. Possible reasons for this phenomenon include the lack of standardized methods for monitoring adverse effects, short study durations that fail to capture delayed reactions, and a primary focus on the ergogenic effects and fatigue recovery of dietary supplements rather than their safety. Future research should adopt more rigorous methodologies to systematically assess and report adverse effects. Furthermore, the five studies that did report adverse reactions primarily concentrated on gastrointestinal issues, neglecting other common side effects. For instance, caffeine is known to cause adverse reactions such as tachycardia, palpitations, nervousness, increased urination, insomnia, and muscle soreness [80]. Adverse events in sports supplementation research are often underestimated due to various methodological limitations. Many trials are short and involve small sample sizes, which limits the ability to observe and detect less common or long-term side effects. Without extended follow-up, delayed or chronic adverse reactions may go unrecognized. In addition, adverse event monitoring is often inconsistent. Some studies rely on passive self-reporting instead of using systematic methods to collect side-effect data, and there is no universally accepted standard for defining or grading supplement-related adverse events [81]. Publication bias also plays a role in underreporting. Studies with no significant results or those that report harmful effects are less likely to be published, which can create a misleading picture that favors positive outcomes. One systematic review found that only 46% of published trial reports included any information on adverse events, compared to 95% of corresponding unpublished reports [81]. This suggests that relying only on published data may lead to an incomplete understanding of the actual risks associated with supplements. These findings show how short study durations, limited sample sizes, and selective reporting can lead to a consistent underestimation of the risks linked to supplement use. Researchers have also noted problems in current reporting practices, such as focusing only on serious events and failing to track adverse effects beyond the main study period [82]. Overall, adverse outcomes in dietary supplement trials are likely more common than the published evidence suggests. This highlights the need for more careful, consistent, and transparent reporting of adverse events in future research.

### 4.4. Interactions Between Different Dietary Supplements

Among the 26 selected studies, Chen et al. (2016) [29], Chou et al. (2018) [3], and Sarshin et al. (2021) [40] evaluated the effects of combined supplementation on ergogenic benefits and recovery from injury. Specifically, their studies examined the impact of branched-chain amino acids, arginine and citrulline, vitamin C and vitamin E, creatine and sodium bicarbonate. Vitamin C and E function as complementary antioxidants, with vitamin E protecting cell membranes from oxidative damage and vitamin C neutralizing free radicals in aqueous environments while also regenerating vitamin E to sustain its protective role [81]. This combination provides a more comprehensive defense against oxidative stress, which is especially important during intense physical activity. Creatine and sodium bicarbonate work together to support anaerobic performance. Creatine enhances adenosine triphosphate production via the phosphagen system, increasing power output, while sodium bicarbonate helps buffer acid accumulation, delaying fatigue and allowing athletes to sustain high-intensity efforts for longer [83]. BCAA, arginine, and citrulline also play a crucial role in managing fatigue and optimizing metabolism [83]. BCAA competes with tryptophan for brain uptake, reducing serotonin production and helping to delay central fatigue [84]. Meanwhile, arginine and citrulline support nitric oxide synthesis and the urea cycle, facilitating ammonia clearance and preventing its toxic accumulation from increased BCAA metabolism [84]. By enhancing energy production, buffering metabolic byproducts, and reducing oxidative stress, these supplement combinations contribute to improved performance, endurance, and recovery in high-intensity sports.

While the combined use of dietary supplements can improve performance, endurance, and recovery, it also introduces the possibility of interactions that may limit benefits or create risks. These interactions can be grouped into several types based on their effects. The first type involves physiological overlap, where two or more supplements influence the same system. For example, combining multiple buffering agents such as beta-alanine and sodium bicarbonate may enhance acid-buffering capacity [85]. However, they may also increase the likelihood of side effects such as gastrointestinal discomfort or muscle tingling, particularly if dosing is not adjusted to individual tolerance [86]. Similar concerns arise with the combined use of caffeine and sodium bicarbonate, which have both been associated with stomach discomfort. When used together, the risk of gastrointestinal symptoms such as nausea or bloating appears to increase, potentially interfering with exercise performance rather than enhancing it [87]. The second type of interaction involves nutrient absorption and metabolism. For instance, vitamin D improves calcium absorption in the small intestine, which supports bone and muscle health. However, when taken with calcium supplements in high doses or over long periods, the combination may increase the risk of hypercalcemia [88]. In addition, other nutrients may interfere with one another’s absorption. The third type of interaction concerns functional interference, where one supplement reduces or cancels out the benefits of another. For example, some evidence suggests that chronic caffeine use may diminish the effectiveness of creatine supplementation in improving muscular strength or power [87]. This may be due to overlapping effects on calcium handling in muscle cells or competition for intracellular transport processes. In such cases, the combination may offer no additional benefit or even result in a smaller improvement than either supplement used alone. These interaction types highlight the importance of a systematic approach to supplement use. Athletes and coaches should evaluate the potential benefits of supplement combinations and consider whether they share similar metabolic pathways, side effect profiles, or absorption mechanisms. Using supplements without considering these factors may lead to reduced effectiveness, increased side effects, or long-term health risks.

### 4.5. Methodological Considerations

Among the included studies, nine had fewer than 15 participants [16,18,20,26,27,28,29,31,37]. Such small sample sizes limit statistical power, which may affect the stability of the observed effects and reduce the reliability of conclusions regarding the supplements. This issue is particularly evident in the studies of Miraftabi et al. (2021) [28] and Lopes-Silva et al., 2018 [37], which included only eight and nine participants, respectively. Both studies used a crossover design, which can help reduce variability by allowing participants to serve as their controls. However, this design also has limitations. If the washout period is too short, the effects of the first intervention may carry over into the next phase and influence the results. Crossover designs are also less appropriate for interventions with long-lasting effects. To improve future research, larger-scale and multi-center studies are needed to enhance statistical reliability, improve representativeness, and reduce differences between studies.

Moreover, many of the included studies (except for those of Kashani et al. (2022) [38] and Riera et al. (2013) [35]) showed an unclear risk of bias, especially regarding random sequence generation and allocation concealment. These methodological issues may have led to an overestimation of the effectiveness of some dietary supplements. For example, if allocation concealment is inadequate, group assignments might be predictable, increasing the chance of selection bias and resulting in systematic differences between groups before the intervention even begins. Such imbalances can interfere with the accurate assessment of treatment effects. Previous research has also indicated that studies using flawed randomization methods tend to report larger treatment benefits than those with proper procedures in place [89]. These limitations point to the need for caution when interpreting the findings and highlight the importance of improving research design in future studies. Using clear randomization methods, maintaining proper allocation concealment, and following reporting standards such as the CONSORT guidelines are key to reducing bias and improving the quality of evidence. Applying these standards for studies focused on dietary supplements in Taekwondo will be essential for generating reliable results that can truly inform training strategies and support evidence-based use of supplements in performance enhancement.

### 4.6. Limitations of the Study

This systematic review is the first comprehensive evaluation of dietary supplement efficacy in taekwondo. Although substantial evidence supports the ergogenic benefits of caffeine, the performance-enhancing effects of other supplements remain unclear, particularly when considering athlete skill levels and sex differences. Robust data on the influence of dietary supplements on injury recovery, immune function, and psychological well-being in taekwondo athletes are lacking. Furthermore, most studies do not document adverse reactions linked to dietary supplement use, which hampers the understanding of their safety profile. Provided that potential side effects can significantly influence athletic performance, dosages that minimize adverse reactions must be employed. Although this review excluded gray literature to ensure a focus on peer-reviewed and methodologically rigorous studies, we acknowledge that this decision may have introduced publication bias by missing relevant data from studies with non-significant or inconclusive results [90]. As a result, the effectiveness of some supplements might have been overestimated. While the performance and recovery effects of many supplements included in this review, such as caffeine, vitamins, and amino acids, are supported by a relatively large body of evidence in other athletic populations, octacosanol and Fufang Ejiaojiang have been far less studied. For these less-investigated supplements, excluding gray literature may have further limited the available evidence and narrowed the interpretive scope of our findings. A more inclusive literature strategy may be considered in future research depending on the research question and available resources.

## 5. Conclusions

This systematic review aimed to present an overview of the effects of dietary supplements on athletic performance and sports injury recovery in taekwondo athletes. Although substantial evidence suggested that caffeine enhanced athletic performance and psychophysiological responses in taekwondo athletes, further research is needed to determine whether other dietary supplements could similarly improve performance, support exercise recovery, and alleviate psychological fatigue. The insights derived from the included studies offer practical guidance for taekwondo practice and competition, thereby contributing to the optimization of nutritional plans, athletic performance, and injury recovery in the competitive taekwondo sport. Future studies should focus on high-quality RCTs, the combined effects of dietary supplements, and differences in response based on sex and training level. Long-term safety remains unclear; so, monitoring potential side effects and interactions is essential.

## Figures and Tables

**Table 1:** Studies on the use of dietary supplements in taekwondo sports.

Study	Dietary Supplement	Dosage	Duration	Participants (Sex)	Physical Characteristics	Level	Results
de Oca et al. (2013) [31]	Creatine	3.5 g/day	6 weeks	10 (men)	20.0 ± 2.0 years	Amateur	Increased fat mass (*p* < 0.028) and elevated serum triglyceride concentration (*p* < 0.037)
					169.0 ± 6.0 cm		
					67.0 ± 9.8 kg		
Riera et al. (2013) [35]	Nucleotide	480 mg/day	30 days	20 (men)	21.4 ± 6.3 years	-	Lymphocyte levels were higher (*p* < 0.0028)
					178.1 ± 8.5 cm		
					73.86 ± 12.6 kg		
Santos et al. (2014) [17]	Caffeine	5 mg/kg	Once	10 (men)	24.9 ± 7.3 years	-	Improved reaction time (*p* = 0.004), elevated plasma lactate concentration (*p* = 0.029 and *p* = 0.014), and no significant change in combat intensity (*p* > 0.05)
					175.0 ± 6.0 cm		
					77.2 ± 12.3 kg		
Lopes-Silva et al. (2015) [18]	Caffeine	5 mg/kg	Once	10 (men)	21.0 ± 4.0 years	International and national levels	Increased estimated glycolytic energy contribution (*p* = 0.04)
					180.0 ± 8.0 cm		
					71.0 ± 12.9 kg		
Chen et al. (2016) [29]	Branched-chain amino acids	0.17 g/kg branched-chain amino acids, 0.05 g/kg arginine and 0.05 g/kg citrulline	Once	12 (men)	20.0 ± 0.8 years	International and national levels	Mitigated central fatigue in exercise
					177.0 ± 4.0 cm		
					66.9 ± 5.0 kg		
Chou et al. (2018) [3]	Vitamin	2000 mg/day Vitamin C and 1400 U/day Vitamin E	4 days	18 (men)	Supplement group: 21.0 ± 0.3 years	International and national levels	Myoglobin levels (*p* = 0.021), plasma creatine kinase activity (*p* = 0.017), and hemolysis (*p* = 0.034) were observed to be lower.
					175.8 ± 2.1 cm		
					67.9 ± 3.0 kg		
					Placebo group:		
					21.3 ± 0.6 years		
					178.1 ± 2.7 cm		
					71.5 ± 3.1 kg		
Jung et al. (2018a) [24]	Vitamin	5000 IU/day Vitamin D	4 weeks	44 (men = 26, women= 18)	20.1 ± 0.15 years	-	Anaerobic peak power (*p* = 0) and isokinetic knee extension strength were improved (*p* = 0.01).
					177.4 ± 1.36 cm		
					71.0 ± 2.07 kg		
Jung et al. (2018b) [25]	Vitamin	5000 IU/day Vitamin D_3_	4 weeks	25 (men)	19.9 ± 1.85 years	-	Reducing the symptoms of upper respiratory tract infection during winter training
					181.7 ± 1.17 cm		
Lopes-Silva et al., 2018 [37]	Sodium bicarbonate	300 mg/kg	-	9 (women)	19.4 ± 2.20 years	National level	Lactate concentration significantly increased (*p* = 0.04), glycolytic energy system contribution was enhanced (*p* = 0.01), and total attack time was extended (*p* = 0.05)
					179.3 ± 3.50 cm		
					70.4 ± 8.9 kg		
Gaamouri et al. (2019) [30]	Carob	40 g/day	6 weeks	23 (men = 12, women = 11)	21.9 ± 1.20 years	National level	Body weight was reduced (*p* < 0.001), and aerobic capacity was improved
					164.0 ± 3.00 cm		
					67.4 ± 17.3 kg		
Lee et al. (2019) [36]	Octacosanol	40 mg/day	6 days	26 (men)	Supplement group:	National level	Improved lipid profiles and mitigated oxidative stress
					18 ± 1.0 years		
					174.7 ± 1.4 cm		
					66.9 ± 6.7 kg		
					Placebo group:		
					18 ± 1.0 years		
					173.9 ± 6.3 cm		
					66.7 ± 5.7 kg		
Wang and Song (2020) [33]	Fufang Ejiaojiang	60 mL/day	2 months	90 (men and women)	Supplement group:	-	Enhanced antioxidant capacity, improved blood oxygen transport ability, boosted aerobic exercise performance and overall athletic capability, and alleviated exercise-induced fatigue
					21.13 ± 1.65 years		
					179.65 ± 1.65 cm		
					70.45 ± 2.69 kg		
					Placebo group:		
					21.25 ± 1.71 years		
					177.91 ± 6.34 cm		
					71.43 ± 2.78 kg		
Antoniett et al., 2021 [26]	Beetroot	1 g beetroot extract	Once	12 (men)	26.8 ± 8.80 years	-	Absolute VO_2Peak_ (*p* = 0.048) and absolute VO_2max_ (*p* = 0.044) were higher
					180.0 ± 10.00 cm		
					77.8 ± 11.7 kg		
Khosravi et al. (2021) [27]	Beetroot	140 mL/day beetroot juice	6 days	12 (men)	19.2 ± 1.60 years	National level	It enhances bilateral muscle strength during isokinetic contractions and reduces fatigue during knee extensor contractions
					181.5 ± 9.20 cm		
					66.4 ± 9.20 kg		
Miraftabi et al. (2021) [28]	Beetroot	60 mL or 120 mL beetroot juice	-	8 (men)	20 ± 4 years	-	Improved cognitive function with 60 mL beetroot juice (*p* < 0.05)
					180 ± 2 cm		
					64.8 ± 4.0 kg		
Sarshin et al. (2021) [40]	Creatine and sodium bicarbonate	20 g/day creatine or 0.5 g/kg/day sodium bicarbonate or 20 g/day creatine and 0.5 g/kg/day sodium bicarbonate	5 days	40 (men)	21 ± 1 years	National level	Both creatine and sodium bicarbonate alone improved performance in the Taekwondo Anaerobic Intermittent Kick Test. However, co-ingestion of CR and SB further enhanced mean power compared to either supplement alone
					180.5 ± 7.3 cm		
					72.7 ± 8.6 kg		
Ghojazadeh et al. (2022) [32]	Curcumin	4 g/day	5 days	18 (men)	22.27 ± 0.94 years	Provincial level	Reduced levels of creatine kinase, lactate dehydrogenase, and malondialdehyde, along with an increase in total antioxidant capacity (*p* < 0.05)
					180.05 ± 4.87 cm		
					74.10 ± 8.67 kg		
Kashani et al. (2022) [38]	Spirulina	8 g/day	3 weeks	18 (men)		-	Reduced plasma levels of lactate dehydrogenase, creatine kinase, and interleukin-6 while significantly increasing plasma total antioxidant capacity, superoxide dismutase, and glutathione peroxidase levels (*p* < 0.05)
Ouergui et al. (2022) [19]	Caffeine	3 mg/kg	-	20 (men = 10, women = 10)	17.5 ± 0.7 years	-	The combination of caffeine ingestion (3 mg·kg^−^^1^) and conditioning activity significantly improved agility (*p* < 0.001), kicking speed (*p* < 0.001), and psychological states (*p* < 0.05)
					168 ± 9 cm		
					59.2 ± 10.0 kg		
Sun et al. (2022) [20]	Caffeine	200 mg	-	10 (men = 6, women = 4)	Male:	National level	The reaction time in the Eriksen Flanker Test decreased (*p* = 0.035), and the peak power and mean power per unit of body weight increased during the Wingate Anaerobic Test (*p* = 0.018 and 0.042)
					27 ± 2 years		
					174 ± 7 cm		
					71 ± 11 kg		
					Female:		
					25 ± 3 years		
					162 ± 6 cm		
					58 ± 1 kg		
Delleli et al. (2023) [21]	Caffeine	3 mg/kg	-	16 (men)	18.25 ± 0.75 years	National level	In the taekwondo-specific agility test, 10 s frequency speed of kick test, and multiple frequency speed of kick test, the performance under the caffeine combined with music condition was significantly better than that under the other conditions (*p* < 0.05)
					182 ± 6.84 cm		
					60.92 ± 8.96 kg		
Ouergui et al. (2023) [13]	Caffeine	3 mg/kg	-	52 (men = 26, women = 26)		-	Elite female athletes demonstrated significantly better performance in the taekwondo-specific agility test, 10 s frequency speed of kick test, and multiple frequency speed of kick test compared to sub-elite female athletes (*p* < 0.001). Elite male athletes showed significantly better performance in the 10 s frequency speed of the kick test (*p* = 0.003) and multiple frequency speed of the kick test (*p* < 0.01) compared to sub-elite male athletes
Zhu et al., (2023) [39]	Yogurt	Up to 250 mL/day	8 weeks	51 (women)	Supplement group:	International level	The Athlete Burnout Questionnaire scores were higher (*p* < 0.05)
					22.10 ± 0.88 years		
					Placebo group:		
					22.50 ± 0.83 years		
Delleli et al. (2024a) [22]	Caffeine	3 mg/kg	-	16 (women)	17–19 years	-	The caffeine with music condition significantly reduced skip and pause times while increasing attack time (*p* < 0.05). It also led to a significant increase in the number of single attacks, combined attacks, counter-attacks (*p* < 0.001), and defensive actions (*p* < 0.05). The mean and peak heart rates were also lower under the CAF + M conditions (*p* < 0.05). Post-combat, the CAF + M condition resulted in higher levels of felt arousal, emotional ratings, and physical enjoyment, while perceived exertion was lower (*p* < 0.05)
					152–187 cm		
					43–59 kg		
Delleli et al. (2024b) [23]	Caffeine	3 mg/kg	-	16 (women)	17.69 ± 0.60 years	-	The condition combining caffeine with music showed the most superior performance in the 10 s frequency speed of the kick test and its multiple versions. Additionally, it elicited the most favorable psychophysiological responses, including perceived exertion, physical enjoyment, feeling scale, and felt arousal scale (*p* < 0.05)
					166.81 ± 10.12 cm		
					49.75 ± 5.36 kg		
Karizak, et al., (2024) [34]	Ginger	2000 mg/day	24 days	24 (women)	19.75 ± 2.03 years	Semi-professional	Serum levels of prostaglandin E2 (*p* < 0.001), cyclooxygenase-2 (*p* = 0.003), and interleukin-6 (*p* = 0.006) demonstrated a significant reduction
					160 ± 6 cm		
					53.99 ± 7.49 kg		

## Data Availability

Data will be made available upon request.

## Supplementary Materials

The following supporting information can be downloaded at: https://www.mdpi.com/article/10.3390/life15040559/s1. Table S1: PRISMA checklist.
